# Dietary Supplementation of 25-Hydroxyvitamin D_3_ Improves Growth Performance, Antioxidant Capacity and Immune Function in Weaned Piglets

**DOI:** 10.3390/antiox11091750

**Published:** 2022-09-03

**Authors:** Xingjian Zhou, Youwei Zou, Youhan Xu, Zeyu Zhang, Yujun Wu, Jindang Cao, Baoqin Qiu, Xiaoyu Qin, Dandan Han, Xiangshu Piao, Junjun Wang, Jinbiao Zhao

**Affiliations:** 1 State Key Laboratory of Animal Nutrition, College of Animal Science and Technology, China Agricultural University, Beijing 100193, China; 2 Shandong Haineng Bioengineering Co., Ltd., Rizhao 276800, China

**Keywords:** weaned piglets, vitamin D_3_, 25-hydroxyvitamin D_3_, antioxidant capacity, intestinal barrier, bone quality

## Abstract

This study was conducted to evaluate the effects of 25-hydroxyvitamin D_3_ (25(OH)VD_3_) and Vitamin D_3_ (VD_3_) supplemented in the diet of weaned piglets on their growth performance, bone quality, intestinal integrity, immune function and antioxidant capacity. A total of 192 weaned piglets were allocated into four groups and they were fed a control diet containing 2000 IU VD_3_ (negative control, NC), NC + 100 ppm colistin sulfate (positive control, PC), NC + 2000 IU VD_3_ (VD_3_) and NC + 2000 IU 25(OH)VD_3_ (25(OH)VD_3_)_._ The results showed that 25(OH)VD_3_ improved the growth performance, bone quality and antioxidase activity of piglets compared with the other groups. Meanwhile, 25(OH)VD_3_ up-regulated ileal mRNA expressions of tight junction proteins and host defense peptides. The VD_3_ group had an increased intestinal sIgA content and mRNA expression of pBD-1 compared with the NC group. Both groups of VD_3_ and 25(OH)VD_3_ altered the microbial β-diversity compared with the NC group, and 25(OH)VD_3_ increased ileal concentrations of acetate and butyrate. In conclusion, our findings indicated that a regular dosage of 2000 IU VD_3_ in the weaned piglets’ diet did not achieve optimal antioxidant capacity and immune function. 25(OH)VD_3_ had better growth performance than VD_3_ at the same inclusion level, which is associated with the improved intestinal integrity and antioxidant capacity.

## 1. Introduction

Changes in the feeding environment and dietary composition for piglets at weaning result in the disturbance of intestinal microbiota and the suppression of immune function, which in turn leads to severe diarrhea incidence and low growth performance [1]. In the past decades, antibiotics have been used widely to alleviate weaning stress and improve weaned piglets’ performance. However, more and more evidence suggests a positive relationship between antibiotic abuse and the pathogen resistance or food safety of animal products [2,3,4,5,6]. Therefore, it is inevitable and urgent for seeking strategies to decrease the use of antibiotics and improve the efficiency of swine production.

Many antibiotic alternatives have been developed for promoting growth performance and enhancing intestinal health for weaned piglets, such as Chinese herb extracts, organic acids, probiotic, eubiotic, synbiotic and antimicrobial peptides [7,8,9,10]. In recent years, with the increased understanding of vitamin D_3_ (VD_3_), the biological functions of VD_3_ on the development of the host immune system, antioxidant capacity and bone quality have been gradually discovered, in addition to regulating calcium and phosphorus metabolism, while a lack of VD_3_ in dietary treatments would lead to weaned piglets having their the growth performance and bone development slowed down, and then suffering rickets or osteomalacia in contrast [11,12,13,14]. VD_3_ can modulate intestinal health by regulating the expression of the vitamin D receptor (VDR), which was found to be highly expressed at the intestine, where the vitamin D signaling promotes innate immunity and maintains the intestinal integrity [15,16]. The intestinal health of weaned piglets is closely related to their intestinal epithelial and microbial composition. The administration of antibiotics, which are often used during the post-weaning period, could impact intestinal microorganism abundance and cause a severe disruption in the piglet gut microbiota ecosystem [17,18]. Additionally, dietary VD_3_ deficiency aggravates the inflammatory response of the intestinal tract to infection, encourages the colonization of the pathogenic bacteria *Citrobacter rodentium* and damages the defense of the host [19]. In practice, the commercial feed of weaned piglets contains 2000 IU vitamin D, and farmers in China always add antibiotics to the commercial feed to prevent pig diarrhea. The potential of extra vitamin D to replace antibiotics has been unclear when weaned piglets were fed a commercial diet.

25-hydroxyvitamin D_3_ (25(OH)VD_3_) is the active metabolite of VD_3_ in vivo, which has a stronger biological activity than VD_3_. 25(OH)VD_3_ is more easily absorbed by the intestine and is more efficient than VD_3_. The supplementation of 25(OH)VD_3_ shortens the metabolic process of VD_3_ in the liver and avoids the loss of bioavailability in the intestine and liver [20,21]. The affinity of 25(OH)VD_3_ for vitamin D-binding proteins is more than 500 times stronger than that of vitamin D_3_ [22]. Previous studies suggested that 25(OH)VD_3_ is one- to three-fold more potent than vitamin D_3_ in sows and piglets’ diets [23,24]. The differences in the actions of 25(OH)VD_3_ and VD_3_ on weaned piglets’ nutrition have been unclear. In addition, the recommended inclusion dosage of VD_3_ for weaned piglets is about 2000 IU to meet their nutrient requirement in commercial pig production [25,26]. However, few studies have been conducted to explore if more VD_3_ or 25(OH)VD_3_ can achieve a better performance of bone quality and immune function and intestinal health for weaned piglets.

We hypothesized that 25(OH)VD_3_ has stronger beneficial effects on the growth performance, immune function, antioxidant capacity and bone quality of weaned piglets compared with VD_3_ or the specific antibiotics, and that a dietary supplementation of extra VD_3_ and 25(OH)VD_3_ for weaned piglets has beneficial effects on immune function and antioxidant capacity compared with the recommended inclusion level of VD_3_. Therefore, this study was conducted to evaluate the effects of VD_3_ or 25(OH)VD_3_ supplemented in the diet containing 2000 IU VD_3_ on the growth performance, bone quality, immune function and antioxidant capacity of weaned piglets.

## 2. Materials and Methods

### 2.1. Ethics Statement

This animal study was approved by the Care and Use of Experimental Animals Committee of the China Agricultural University (AW20602202-1-2) in 2022. All protocols were performed according to the relevant standards of animal welfare of China Agricultural University.

### 2.2. Animals, Diets and Feeding Management

A total of 192 weaned piglets (Duroc × Landrace × Yorkshire) provided by 26 lactating sows with an initial body weight (BW) of 8.01 ± 0.43 kg were selected and randomly divided into 4 treatment groups with 6 replicates and 8 piglets (4 boars and 4 gilts) per replicate, and the piglets in the one pen were from different litters of lactating sows. Dietary treatments included a negative control diet containing 2000 IU VD_3_ (NC), NC + 100 ppm colistin sulfate (PC), NC + 2000 IU VD_3_ (VD_3_) and NC + 2000 IU 25(OH)VD_3_ (25(OH)VD_3_). A premix of vitamins and trace minerals were supplemented into the piglets’ diets to meet the nutrient requirements of the weaned pigs (NRC, 2012) [27]. The ingredient compositions and nutritive levels of the NC diet are shown in Table 1. The experiment lasted for 42 d. The feeding trial was completed at the FengNing Swine Research Unit of China Agricultural University (Chengdejiuyun Agricultural and Livestock Co., Ltd., Hebei, China). There were no chronic health challenges in the herd during the experimental period. The piglets were provided ad libitum access to water and crumble diets. The weaned piglets were housed in a pen (1.5 m × 1.2 m × 0.8 m). The humidity and temperature of the feeding room for the weaned piglets were controlled at 50~60% and 25~28 °C. 

### 2.3. Sample Collection

On d 28, one piglet from each pen that was close to the average BW of the pen was selected to collect a 10 mL blood sample using the anterior vena cava, which was then centrifuged at 3000 rpm (rounds per minute) and 4 °C for 15 min to collect serum for the further analysis. The selected piglets were slaughtered to collect mucosal and digesta samples of the ileum and colon, and the samples were frozen in liquid nitrogen and stored at −80 °C. In addition, 3 samples of colonic digesta from each treatment were randomly selected for microbial analysis. The femur and tibia of the hind leg were collected and stored at −20 °C. About 2 cm of the ileum and colon were taken and fixed in a 4% paraformaldehyde solution for H&E (hematoxylin-eosin) and PAS (Periodic Acid-Schiff) staining. 

### 2.4. Chemical Analysis

The diets were determined for gross energy (GE), dry matter (DM) (Method 934.01), ether extract (EE) (Method 920.39), crude protein (CP)(Method 990.03) and chromium (Method 990.08) in duplicate [28]. The concentrations of short-chain fatty acids (SCFAs) in the intestinal digesta were determined according to a previous report [29,30]. Briefly, 0.5 g of freeze-dried digesta samples were weighed in a 10 mL centrifuge tube and 8 mL of MilliQ water (Millipore, MA, USA) was added. The samples were centrifuged at 5000 rpm for 10 min after 30 min of sonication, and the supernatant was diluted 50 times and filtered through a 0.22 µm filter membrane (Millipore, MA, USA). The sample solution was analyzed by ion chromatography (DIONEX ICS-3000, Sunnyvale, CA, USA).

### 2.5. Growth Performance and Diarrhea Incidence

On d 0, 14, 28 and 42, the piglets’ BW and their feed consumed were weighed to determine the average daily feed intake (ADFI) and average daily gain (ADG). The feed conversion ratio (FCR) of the weaned piglets was calculated as a ratio of the ADG to ADFI. The piglets were observed for diarrhea situations by a visual assessment method. The diarrhea score was recorded daily and the diarrhea rate was calculated following the method recommended by our labs [31]. The piglets were observed for clinical signs of diarrhea every day, and a scoring system was applied to indicate the presence and severity of the diarrhea as follows: 1 = hard feces; 2 = slightly soft feces; 3 = soft, partially formed feces; 4 = loose, semiliquid feces; and 5 = watery, mucous-like feces.

Diarrhea incidence (%) = total number of piglets with diarrhea/(number of piglets × number of experiment days) × 100.

### 2.6. Bone Quality

The bone density was determined according to Archimedes’ principle: Bone density = [A/(A-B)] × P, where A is the weight after leaving the water surface, B is the weight when fully immersed in distilled water and P is the density of the distilled water. The bone mechanical properties were determined by a three-point bending test using an MTS-810 universal tensile tester (MTS Systems Corporation, Eden Prairie, MN, USA) [32]. Briefly, the tibia and femur were placed on a support and continuous pressure was applied to the midpoint of the bone to deform the bone until it broke. The span of the loading point was 30 mm and the loading speed was 10 mm/min. The load–displacement curves were recorded and the fracture strength, damage deflection, stiffness and absorbed energy were calculated. Crude ash, calcium and phosphorus contents were determined as described by a previous report [33]. The bones were wrapped in degreased gauze, boiled in deionized water at 100 °C for 2 h and dried at 55 °C for 24 h. Then, the treated samples were extracted with ether for 96 h and dried at 55 °C and 100 °C for 18 h and 2 h. 

### 2.7. Intestinal Morphology and Globet Cells

The fixed ileum and colon tissue samples were successively dehydrated by the alcohol gradient and they were made transparent with xylene and were embedded in paraffin, and the wax blocks were cooled by a freezing table at −20 °C; then, they were sliced and preserved at room temperature. The intestinal slices were stained with hematoxylin for 7 min; differentiated with a 1% hydrochloric acid–alcohol solution for 5 s; repeatedly washed with distilled water for 15 min; stained with eosin again for 1 min; washed repeatedly with distilled water for 6 min; dehydrated with ethanol, anhydrous ethanol and xylene; covered with glass slides; and dried overnight. The PAS staining was performed according to the following instruction: after routine dewaxing, add 3% acetic acid and incubate at room temperature for 3 min; after removing the acetic acid, add the PAS solution and incubate at 37 °C for 15 min; rinse with 3% acetic acid solution for 10 s; wash repeatedly with distilled water for 4 min; dye with the nuclear fast red solution for another 5 min; wash for 4 min repeatedly with distilled water; and lastly, dehydrate and seal tablets and dry overnight. The villus height, crypt depth and the number of goblet cells were measured by the Zeiss Axio Imager 2 system (Carl Zeiss, Jena, Germany) and at least 10 visual fields were selected for statistics.

### 2.8. Antioxidant Capacity and Immune Function of the Serum and Intestinal Mucosa

About 100 μL of serum or 100 mg of frozen intestinal mucosa were minced and homogenized in 1 mL of pre-chilled RIPA in a protease inhibitor mixture completely free of EDTA (Roche, Penzberg, Germany). The homogenate was centrifuged at 12,000 rpm for 15 min at 4 °C to collect the supernatant. The protein concentrations were determined using a BCA protein assay kit (Thermo Fisher Scientific, MA, USA) and were then diluted to the same concentration for subsequent analysis. 

The MDA (malondialdehyde), sIgA (secreted immunoglobulin A), IgA, IgG, IgM, IL-2 (interleukin 2), IL-6, IL-10, IL-12, IL-1β and TNF-α (tumor necrosis factor α) contents and the activities of SOD (superoxide dismutase), GSH-Px (glutathione peroxidase) and the T-AOC (the total antioxidant capacity) were determined using the commercial ELISA kits (Shanghai Meilian Biotechnology Co., Ltd., Shanghai, China).

### 2.9. Quantification PCR of Intestinal Junction Proteins and Immune Function

The total RNA was extracted from the intestinal tissues by the RNA pure Kit (CWbiotech Co., Ltd, Jiangsu, China) according to the manufacturer’s protocol. The extracted RNA was quantified using NanoDrop 2000 (Thermo Fisher Scientific, MA, USA) and was then diluted to the same concentration. cDNA was obtained from RNA using a reverse transcription kit (Takara, Kusatsu, Shiga, Japan). Quantitative PCR was performed according to the SYBR Premix Ex Taq II instructions (Takara, Kusatsu, Shiga, Japan) on a Riche light cycler 96 Real-Time PCR System (Roche, CA, USA). The primers of the intestinal epithelial tight junction proteins (Claudin-1, Occludin and ZO-1), host defense peptides (pBD-1, pBD-2, PG1-5) and antioxidant factors (SOD, CAT, GSH-Px) were given in Table S1 and were synthesized by Sangon Biotech Co., Ltd. (Shanghai, China). The relative expressions of target genes to that of a housekeeping gene (GAPDH) were calculated by using the 2^−ΔΔCt^ method.

### 2.10. Bacterial Community

The colon digesta were collected and frozen at −80 ℃. The total genomic DNA of the samples was extracted using the QIAamp Fast DNA Stool Mini Kit (Qiagen, Tübingen, Germany). The V3-V4 region of the 16S rRNA gene was amplified using universal primers and was then pooled into equimolar amounts and then sequenced on the Illumina MiSeq platform to generate paired-end reads of 300 bp. UPARSE (version 7.0) was used to cluster the remaining high-quality sequences into OTUs with 97% similarity, and chimeric sequences were removed using UCHIME. The taxonomy assignment of OTUs was conducted with the RDP classifier against the SILVA 16S rRNA gene database (Release132) with a confidence threshold value of 0.70. The data were analyzed on the Majorbio Cloud Platform (www.majorbio.com) until 2 June 2022 [34]. The β-diversity between the microbiomes was calculated by the Bray–Curtis distance. The dominant genus promoted by 25(OH)VD_3_ and VD_3_ supplementations in the piglets’ colon was analyzed by LEfSe (Linear discriminant analysis Effect Size) analysis.

### 2.11. Statistical Analysis

The experimental data were statistically presented in replicates using the GLM model in SAS 9.2 statistical software (Cary, NC, USA). The individual pen was used as a statistical unit for growth performance and the individual pig was used as a statistical unit for the other data in this manuscript. The different dietary treatments were fixed effects and the animal health status and body weight were random effects. The model residuals and the normal distribution with equal variance were tested for outliers by the UNIVARIATE procedure. Tukey’s test was used for multiple comparisons and the results were expressed as least squares means as well as mean standard errors, with *p* < 0.05 as significant differences and 0.05 < *p* < 0.10 as a trend toward differences.

## 3. Results

### 3.1. Effects of 25(OH)VD_3_ and VD_3_ Supplementations on the Growth Performance of Weaned Piglets

The effects of VD_3_ and 25(OH)VD_3_ on the growth performance and diarrhea incidence of weaned piglets are shown in Table 2. Dietary supplementations of 25(OH)VD_3_ improved the ADFI and ADG of weaned piglets compared with NC, PC and VD_3_ treatments on d 14–28, d 28–42 and d 0–42 (*p* < 0.05), but the ADG and ADFI of the piglets were not significantly different among all dietary treatments in d 0–14. The BW of the piglets in the 25(OH)VD_3_ group was significantly greater than that in the NC, PC and VD_3_ groups on d 28 and d 42 (*p* < 0.05). There was no significant difference in the piglets’ FCR and diarrhea incidence among all the dietary treatments.

### 3.2. Effects of 25(OH)VD_3_ and VD_3_ Supplementations on the Bone Quality of Weaned Piglets 

As shown in Table 3, dietary supplementations of 25(OH)VD_3_ significantly increased the calcium content of the tibia and femur in the weaned piglets compared with the NC, PC and VD_3_ treatments (*p* < 0.05). Meanwhile, 25(OH)VD_3_ improved the bone mineral content (BMC) and the breaking strength of the tibia and femur compared with the NC group (*p* < 0.05). In addition, dietary supplementations of 2000 IU VD_3_ improved the BMC and breaking strength of the tibia in weaned piglets compared with the NC and PC groups (*p* < 0.05).

### 3.3. Effects of 25(OH)VD_3_ and VD_3_ Supplementations on the Serum Antioxidant Capacity and Immune Functions of Weaned Piglets 

**Figure 1:**
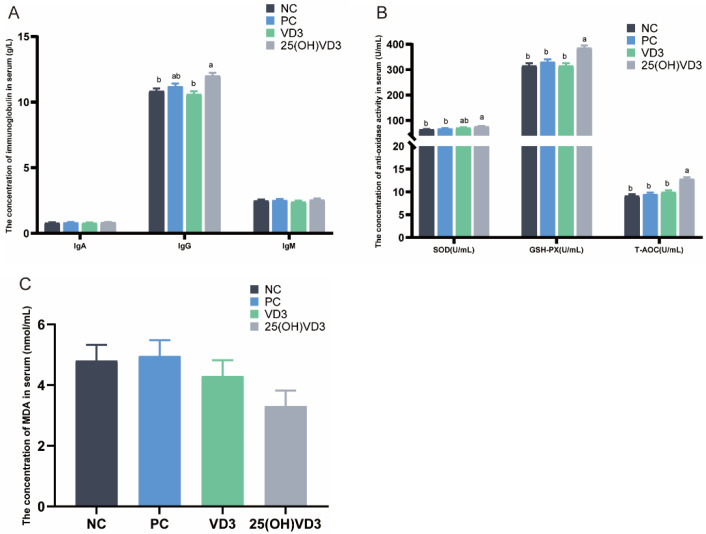
Effect of VD_3_ and 25(OH)VD_3_ on the serum antioxidant capacity of weaned piglets. (**A**): The concentrations of IgA, IgG and IgM in piglets’ serum. (**B**): The concentration of antioxidase activities in serum. (**C**): The concentration of MDA in serum. Note: Different letters on the top of the column indicate significant differences (*p* < 0.05), n = 6. NC, negative control; PC, positive control; VD_3_, vitamin D_3_; 25(OH)VD_3_, 25-hydroxyvitamin D_3_; MDA, Malondialdehyde.

Dietary supplementations of 2000 IU 25(OH) VD_3_ significantly increased the serum content of IgG and the activities of SOD, GSH-Px and the T-AOC in weaned piglets compared with the NC and PC groups (*p* < 0.05) (Figure 1A,B). In addition, 25(OH) VD_3_ increased the serum concentration of IgG and the activities of GSH-Px and the T-AOC compared with the VD_3_ group (*p* < 0.05), and it had a tendency to decrease the serum MDA content when the weaned piglets were fed a 25(OH) VD_3_ diet (Figure 1C). 

### 3.4. Effects of 25(OH)VD_3_ and VD_3_ Supplementations on the Intestinal Morphology and Goblet Cell Numbers of Weaned Piglets 

**Figure 2:**
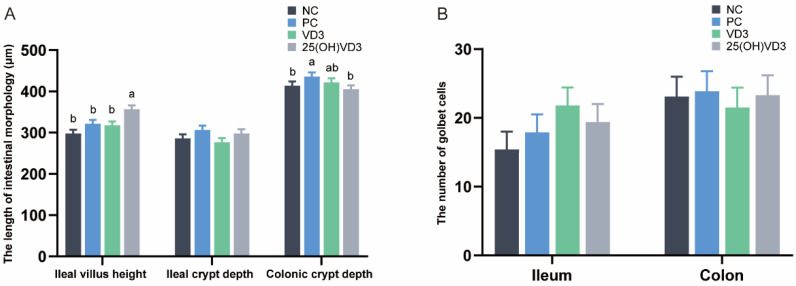
Effect of VD_3_ and 25(OH)VD_3_ on the intestinal morphology of weaned piglets. (**A**): The length of villus height and crypt depth in the piglets. (**B**): The numbers of goblet cells in the ileum and colon. Note: Different letters on the top of the column indicate significant differences (*p* < 0.05), n = 6. NC, negative control; PC, positive control; VD_3_, vitamin D_3_; 25(OH)VD_3_, 25-hydroxyvitamin D_3_.

As shown in Figure 2A, dietary supplementations of 2000 IU 25(OH) VD_3_ increased villus height in the ileum of the weaned piglets (*p* < 0.05), but 25(OH) VD_3_ had no significant effect on the crypt depth of the ileum. The crypt depth in the colon of the piglets in the PC group was greater than that in the NC and 25(OH) VD_3_ groups (*p* < 0.05). In addition, there were no significant differences in the numbers of goblet cells in the ileum and colon of the weaned piglets (Figure 2B). 

### 3.5. Effects of 25(OH)VD_3_ and VD_3_ Supplementations on the Intestinal Immune Function of Weaned Piglets 

**Figure 3:**
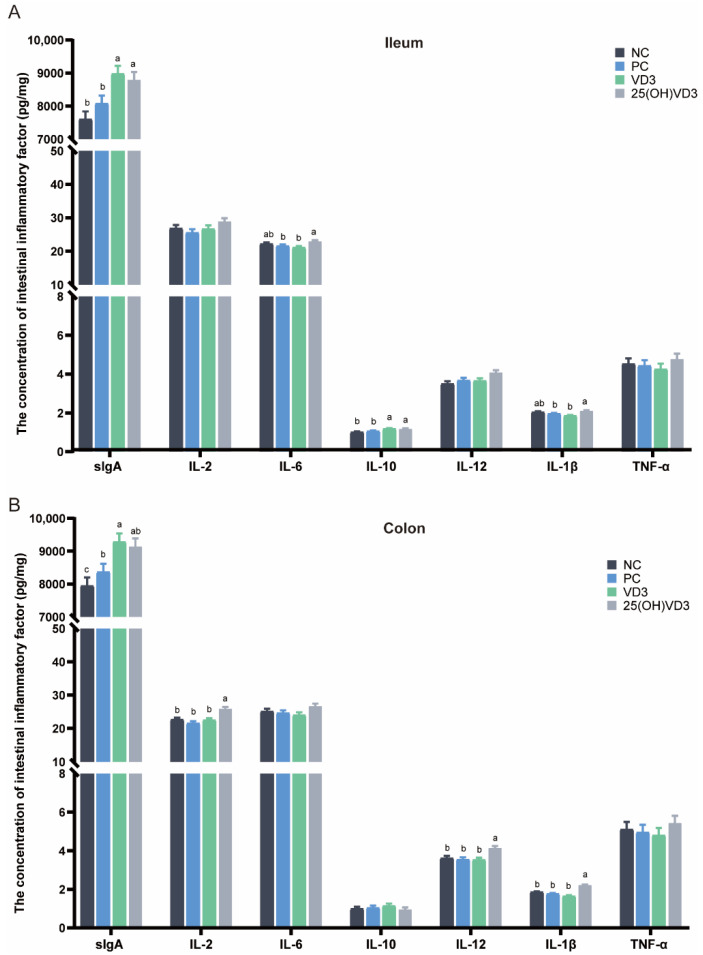
Effect of VD_3_ and 25(OH)VD_3_ on the intestinal immune function of weaned piglets. (**A**): The concentration of intestinal inflammatory factors in the ileum. (**B**): The concentration of intestinal inflammatory factors in the colon. Note: Different letters on the top of the column indicate significant differences (*p* < 0.05), n = 6. NC, negative control; PC, positive control; VD_3_, vitamin D_3_; 25(OH)VD_3_, 25-hydroxyvitamin D_3_.

As shown in Figure 3, dietary supplementations of 2000 IU 25(OH)VD_3_ increased concentrations of sIgA and IL-10 in the ileal mucosa of weaned piglets (*p* < 0.05), and IL-2, IL-12 and IL-1β in the colonic mucosa (*p* < 0.05) compared with the NC and PC groups. Meanwhile, the group of 25(OH)VD_3_ increased concentrations of IL-6 and IL-1β in the ileal mucosa and IL-2, IL-12 and IL-1β in the colonic mucosa compared with the VD_3_ group (*p* < 0.05). In addition, the group of VD_3_ increased the concentrations of sIgA in the ileal and colonic mucosa of the piglets (*p* < 0.05).

### 3.6. Effects of 25(OH)VD_3_ and VD_3_ Supplementations on the Ileal mRNA Expression of Host Defense Peptides, Tight Junction Proteins and Antioxidation Capacity

**Figure 4:**
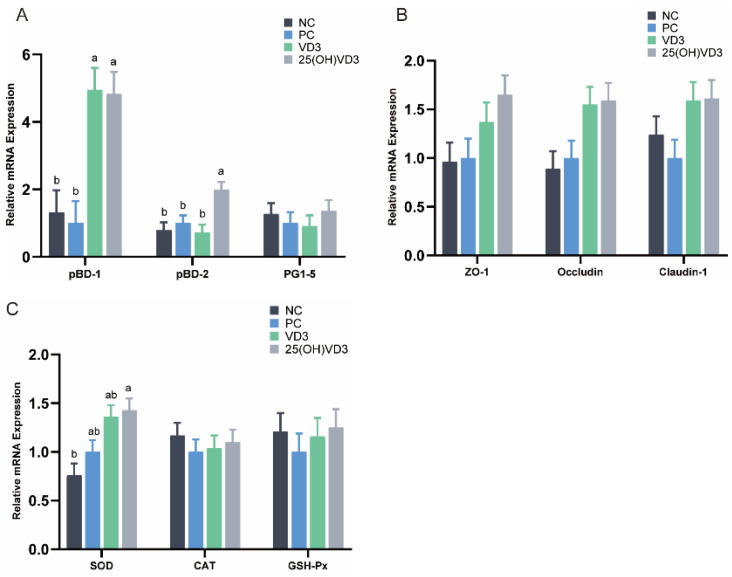
The relative mRNA expressions in the weaned piglets’ ileum. (**A**): The mRNA expression of host defense peptides (pBD-1, pBD-2, PG1-5). (**B**): The mRNA expression of tight junction proteins (ZO-1, Occludin, Claudin-1). (**C**): The mRNA expression of antioxidases (SOD, CAT, GSH-Px). Note: Different letters on the top of the column indicate significant differences (*p* < 0.05), n = 6. NC, negative control; PC, positive control; VD_3_, vitamin D_3_; 25(OH)VD_3_, 25-hydroxyvitamin D_3_.

Dietary supplementations of 2000 IU 25(OH)VD_3_ up-regulated mRNA expressions of pBD-1 and pBD-2 (*p* < 0.05) compared with the NC and PC treatments (Figure 4A). Additionally, there was a tendency for 2000 IU 25(OH)VD_3_ to up-regulate the mRNA expression of Occludin, Claudin-1 and ZO-1 (*p* < 0.10) in the ileum of piglets (Figure 4B). In addition, dietary supplementations of 2000 IU VD_3_ increased the mRNA expression of pBD-1 compared with the NC and PC groups (*p* < 0.05). Meanwhile, 25(OH)VD_3_ increased the mRNA expression of SOD activity in the ileal mucosa of the piglets compared with the NC group (*p* < 0.05) (Figure 4C). 

### 3.7. Effects of 25(OH)VD_3_ and VD_3_ Supplementations on the Composition and Diversity of Colonic Microbial Community

**Figure 5:**
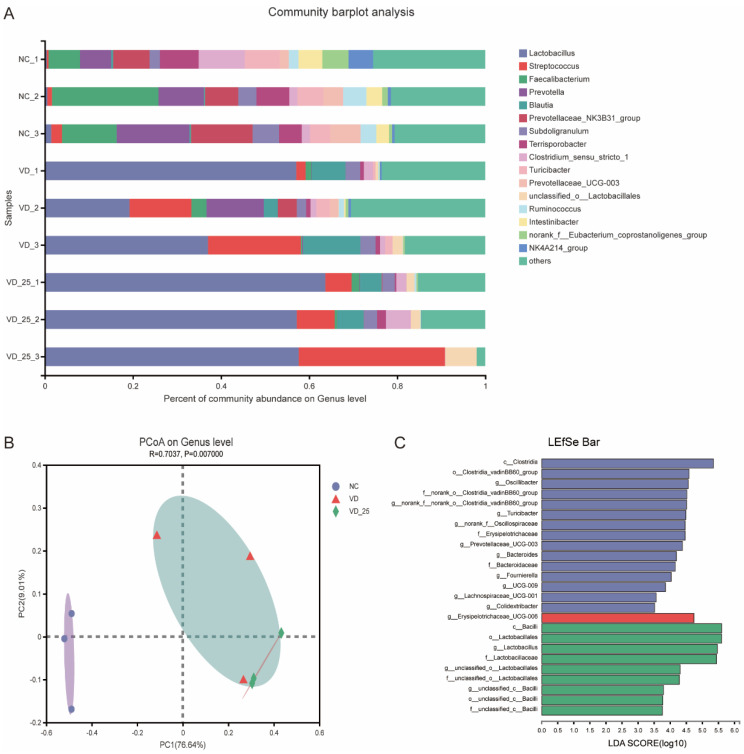
Effect of VD_3_ and 25(OH)VD_3_ on colonic microorganisms of weaned piglets. (**A**): Microbial composition of colon digesta on genus level. (**B**): PCoA of the microbiome based on weighted Bray–Curtis distance metrics. (**C**): The dominant genus promoted by three diets by LEfSe analysis. n = 3; NC, negative control; VD, vitamin D_3_; VD_25, 25-hydroxyvitamin D_3_.

A significant cluster of microbial composition in the colon digesta was observed between NC and VD_3_ or 25(OH)VD_3_ (Figure 5B). Additionally, both groups of VD_3_ and 25(OH)VD_3_ significantly increased an abundance of *Lactobacillus* compared with the NC (*P* < 0.05) (Figure 5A). Further microbial analyses by the LEfSe analysis revealed that populations of *Lactobacillus* and *Bacillus* were greater in the 25(OH)VD_3_ group, and that *Oscillibacter*, *Turicibacter*, *Prevotellaceae_UCG-003* and *Bacteroides* were enriched in the NC group (Figure 5C).

### 3.8. Effects of 25(OH)VD_3_ and VD_3_ Supplementations on Intestinal Concentration of SCFAs

**Figure 6:**
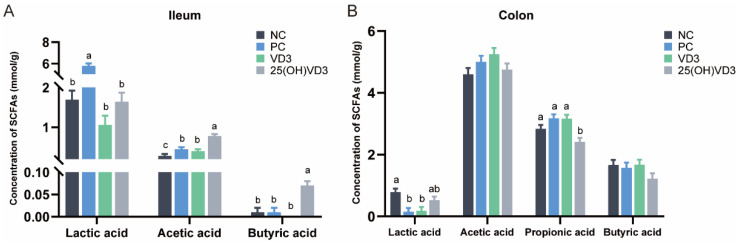
The concentration of SCFAs in the intestinal digesta of weaned piglets. (**A**): The concentration of SCFAs in the ileum. (**B**): The concentration of SCFAs in the colon. Note: Different letters on the top of the column indicate significant differences (*p* < 0.05), n = 6. NC, negative control; PC, positive control; VD_3_, vitamin D_3_; 25(OH)VD_3_, 25-hydroxyvitamin D_3_.

As shown in Figure 6, 25(OH)VD_3_ increased concentrations of acetic acid and butyric acid in the ileal digesta compared with the other dietary groups (*p* < 0.05) but decreased the concentration of propionic acid in the colonic digesta of weaned piglets. Compared with the NC group, dietary supplementations of 2000 IU VD_3_ also increased a concentration of acetic acid in the ileum of the weaned piglets (*p* < 0.05).

## 4. Discussion

25(OH)VD_3_ is a derivative of cholecalciferol vitamin D_3_ in the liver, which has a higher bioavailability than vitamin D_3_. In this study, pig BW, ADFI and ADG on d 28 and d 42 in the group of 2000 IU 25(OH)VD_3_ were greater than the other dietary treatments, indicating that 25(OH)VD_3_ had a stronger positive effect on the improvement of growth performance in the piglets compared with VD_3_ and colistin sulfate. However, there were no significant differences in the ADFI and ADG between the VD_3_ and NC groups, which indicated that the greater dosage of VD_3_ supplemented in the diet cannot improve the growth performance of weaned piglets compared with the regular level of 2000 IU VD_3_. A previous study reported that diets with different inclusion levels of 25(OH)VD_3_ resulted in no significant differences in the BW of weaned piglets, but the ADFI and ADG of the piglets showed a quadratic response as the inclusion levels of 25(OH)VD_3_ increased from 0 to 4000 IU. Additionally, in a previous study, this was the recommended inclusion level of 25(OH)VD_3_ is 2000 IU for achieving a better growth performance [26]. In addition, the reason for the negative effects of excess 25(OH)VD_3_ on pig performance should be clarified in further studies. Furthermore, 25(OH)VD_3_ alleviated the negative effects of the diet containing low calcium and phosphorus contents on the growth performance of the weaned piglets, which indicated that 25(OH)VD_3_ increased the absorption of calcium and phosphorus [35]. A previous study showed that weaned piglets were the most sensitive pigs to the dose of 25(OH)VD_3_ compared with growing-finishing pigs, and no significant modifications related to the dietary treatments of 1000, 2000 and 4000 IU 25(OH)VD_3_ were found in the growth performance and concentrations of calcium and phosphorus in the plasma of weaned piglets [36]. Meanwhile, the results of resistance trails showed that the addition of 25(OH)VD_3_ at 10 times the recommended dose (20,000 IU/kg) did not adversely affect the physiological indices, organ weights, bone parameters and renal calcium content of pigs [37,38], which indicated that the effects of greater inclusion levels of 25(OH)VD_3_ on pig performance should be further explored in our study. Yang et al. also reported that the addition of different doses of 25(OH)VD_3_ to the diet had no significant effect on growth performance in weaned piglets [39]. In addition, there is no difference in pig diarrhea among all treatments, and the reason for the observation above may be associated with the low integral incidence situation of the piglets in the study. However, a previous in vitro study reported that 1,25-Dihydroxyvitamin D3 inhibited porcine epidemic diarrhea virus replication by regulating cell cycle resumption [40], which may be associated with the greatest efficiency of 1,25-Dihydroxyvitamin D3 to improve intestinal barrier function.

The piglets at weaning would suffer the oxidation stress due to changes in diets and the feeding environment, resulting in damage to the intestinal barrier function and the reduced antioxidant capacity for piglets. In this study, the ileal mRNA expression of the SOD and serum activities of GSH-Px, SOD and the T-AOC in the 25(OH)VD_3_ group were significantly greater than those in the NC group, suggesting that dietary 25(OH)VD_3_ supplementation alleviated the oxidative stress in the weaned piglets by improving antioxidase activities. Yang et al. reported that dietary supplementations of 118 μg/kg 25(OH)VD_3_ had positive effects on immune function and antioxidant capacity in weaned piglets [39]. Zhang et al. reported that 50 or 75 μg/kg 25(OH)VD_3_ additionally increased the serum activities of the T-AOC and GSH-Px in weaning piglets [26]. Oxidation stress at weaning always increases serum concentrations of MDA and hydrogen peroxide in piglets, and Yang et al. reported that 43 μg/kg 25(OH)VD_3_ additionally decreased serum MDA content [25,39], which is consistent with our finding. In addition, a previous report showed that VD_3_ mitigated oxidative stress by improving the activities of SOD and GSH-Px [41], but there was no positive response of 2000 IU VD_3_ supplementation on the antioxidase activity of weaned piglets in our study. These results above indicated that dietary supplementations of an extra 25(OH)VD_3_ alleviated the oxidative damage to the piglets at weaning, rather than VD_3_. 

Immunoglobulins are secreted mainly by B cells and reflect the actual humoral immunity of the host [42,43]. Immunoglobulin G (IgG) is a keystone in the immune response by agglutinating and precipitating antigens, or neutralizing toxins and viruses [44]. In the study, the extra addition of 2000 IU 25(OH)VD_3_ increased the serum IgG content compared with the NC and VD_3_ groups, indicating an improved protective humoral and immune function in response to the infection. A previous study also reported that 25(OH)VD_3_ increased IgG concentrations in the serum of piglets after weaning [21]. However, a previous study reported that dietary supplementations of 25(OH)VD_3_ did not affect the serum IgG content for growing-finishing pigs, which may be associated with the developed immune system [45]. In addition, both 25(OH)VD_3_ and VD_3_ increased sIgA and IL-10 concentrations in the ileum compared with the NC group, which indicated that the extra supplementation of 2000 IU VD_3_ improved intestinal immune function in the weaned piglets. Recent evidence also demonstrated that dietary vitamin D_3_ intaking is an immunoregulatory hormone that modulates the innate and adaptive immune system [16]. Overall, our results suggest that the extra addition of 2000 IU 25(OH)VD_3_ or VD_3_ to the diet alleviates the inflammatory response of weaned piglets induced by the oxidation stress.

Previous studies have shown that the intestinal villi and crypt directly affect the intestinal epithelial barrier and absorption function of dietary nutrients [46,47,48]. A ratio of villus height to crypt depth is positively correlated with the ability of the small intestine to absorb nutrients [49]. In the present study, 25(OH)VD_3_ supplementations increased the villus height in the ileum compared with the NC, PC and VD_3_ treatments, which indicated that 25(OH)VD_3_ is beneficial for the development of the small intestine and absorption of nutrients. However, extra supplementations of 2000 IU VD_3_ had no positive effects on intestinal morphology compared with the NC diet. A previous study reported that the inclusion level of 25(OH)VD_3_ showed a quadratic response on intestinal morphology and dietary supplementations of 2000 IU 25(OH)VD_3_ was recommended [26]. In addition, intestinal goblet cells secrete mucins to strengthen intestinal barrier function; however, the current study indicated that both VD_3_ and 25(OH)VD_3_ had no positive effects on the proliferation of intestinal goblet cells. Tight junction proteins are critical for regulating intestinal permeability and maintaining the integrity of the intestinal tissue [50,51]. In our study, both 25(OH)VD_3_ and VD_3_ increased the mRNA expression of tight junction proteins, resulting in an improved intestinal barrier function in the weaned piglets. Many studies have reported that the down-regulation of ZO-1, Occludin and Claudin-1 expression would increase intestinal permeability and lead to intestinal-related diseases [52,53,54]. The intestinal mucosal defense was conducted by the tight junction expression of the intestinal epithelia along with defensins and lysozymes secreted by the Paneth cells, which could be promoted by dietary VD_3_ supplementation [55]. Therefore, 25(OH)VD_3_ and VD_3_ are beneficial for maintaining intestinal integrity due to their positive effects on improving tight junction protein expression, and 25(OH)VD_3_ can also improve the development of intestinal villi compared with the VD_3_.

Many studies have reported that VD_3_ promotes the absorption of calcium and phosphorus, resulting in calcium deposits and improving bone quality [56]. In the present study, dietary supplementations of an extra 2000 IU VD_3_ increased the tibial mineral content of weaned piglets compared with the NC and PC groups, while 25(OH)VD_3_ increased femoral mineral contents compared with the PC group. In addition, both VD_3_ and 25(OH)VD_3_ increased tibial and femoral breaking strength and stiffness in weaned piglets compared with the NC and PC groups, while 25(OH)VD_3_ had a better breaking strength in the femur compared with the VD_3_ group. Zhao et al. reported that 25(OH)VD_3_ supplementations increased the stiffness of femurs when weaned piglets were fed a diet containing low concentrations of calcium and phosphorus [35]. Previous publications reported that serum VD_3_ concentration and bone mineral content increased when sows were fed a 2000 IU 25(OH)VD_3_ diet, and that 25(OH)VD_3_ supplementation promoted the bone quality of tibias and femurs in suckling piglets [26,57]. It has been reported that the positive effects of 25(OH)VD_3_ on calcium deposit and bone quality are associated with the up-regulated expression of vitamin D receptor (VDR) signaling [33,58]. Oxidation stress at weaning for weaned piglets would disturb the gut microbiota community due to changes in diets and the feeding environment. The consequence of microbial disturbance is the decreased abundance of beneficial bacteria, such as *Lactobacillus*, which produce lactic acid and SCFAs to decrease intestinal pH and suppress the growth of harmful bacteria [59,60]. *Lactobacillus* is characterized as one of the probiotics by their ability to colonize the intestinal mucus layer and to produce antimicrobial substances to suppress the growth of harmful bacteria, such as bacteriocin, organic acids and hydrogen peroxide [61,62,63,64]. A significant cluster of microbial compositions in the colon digesta was observed between the NC and VD_3_ or 25(OH)VD_3_ group in our study_,_ indicating that both 25(OH)VD_3_ and VD_3_ had a similar response to the microbial structure and composition compared with NC. The intestinal VDR also plays a fundamental role in intestinal homeostasis through its effects on autophagy [16]. A previous study reported that the intake of VD_3_ regulated gut microbiota and protected mice from dextran sodium sulfate-induced colitis [65]. Bashir et al. reported that VD_3_ supplementation decreased the relative abundance of *Escherichia-Shigella* and increased microbial richness in the intestine [12]. There are mutual interactions between 25(OH)VD_3_, the intestinal barrier and the microbiome that still remain to be fully understood. Our study suggested that extra VD_3_ and 25(OH)VD_3_ supplementations may represent a master regulator of inflammation by promoting calcium absorption and regulating the production of antimicrobial peptides (pBD-1 and pBD-2) that, in turn, were responsible for remodeling the bacterial communities that comprised the intestinal microbiota. Our results also showed that the dietary group of 25(OH)VD_3_ increased a relative abundance of *Lactobacillus* in the colon of the weaned piglets, which may provide a view that 25(OH)VD_3_ enriched the intestinal abundance of *Lactobacillus* throughout the intestinal health. The SCFAs are metabolites of gut microbiota, mainly including acetic acid, propionic acid and butyric acid. Acetic acid and propionic acid are absorbed to provide energy for the cellular metabolic activities of the host, while butyric acid as a source of energy for epithelial cells increases the number of goblet cells in the jejunum and ileum [66,67]. In our study, concentrations of acetic acid and butyric acid in the ileal digesta were higher in the 25(OH)VD_3_ group than those in the NC and PC groups. However, a previous study reported that VD_3_ supplemented in diets containing different dietary calcium and phosphorus levels did not affect intestinal SCFAs concentrations [68]. Therefore, the role of VD_3_ and 25(OH)VD_3_ in regulating microbial composition and structure in the intestine of weaned piglets should be further explored.

## 5. Conclusions

Extra supplementations of 2000 IU VD_3_ or 25(OH)VD_3_ based on a diet containing 2000 IU VD_3_ is beneficial to the immune function and bone quality of weaned piglets by increasing intestinal sIgA concentration and bone mineral content and breaking strength. In comparison with VD_3_, dietary supplementations of 25(OH)VD_3_ improved the antioxidant capacity to alleviate the oxidation stress of the weaned piglets, resulting in an improved growth performance and intestinal barrier function. Above all, 25(OH)VD_3_ can be used as functional antibiotics alternative to improve growth performance via enhancing the bone quality, antioxidant capacity and immune function of weaned piglets. 

## Figures and Tables

**Table 1:** Composition and nutrient levels of the basal diet (%, as-fed basis).

Items	d 1~14	d 15~42
Ingredients %		
Corn	57.11	63.78
Soybean meal	16.00	18.00
Extruded full-fat soybean	14.00	10.00
Fishmeal	4.00	2.00
Whey powder	4.00	2.00
Soybean oil	1.55	0.92
Dicalcium phosphate	1.00	0.87
Limestone powder	0.82	0.90
Salt	0.30	0.30
L-Lysine HCl	0.45	0.45
DL-Methionine	0.09	0.10
L-Threonine	0.15	0.15
L-Tryptophan	0.03	0.03
Premix ^1^	0.50	0.50
Analyzed nutrient level %		
Crude protein	19.36	18.01
Calcium	0.82	0.75
Total phosphorus	0.61	0.53
Calculated nutrient level		
Digestible energy(MJ/kg)	13.55	13.50
Metabolizable energy (MJ /kg)	12.82	12.74
SID Lys	1.35	1.23
SID Met	0.39	0.36
SID Thr	0.79	0.73
SID Trp	0.22	0.20

Note: ^1^ premix provides: Vitamin A, 12,000 IU; Vitamin D_3_, 2000 IU; Vitamin E, 30 IU; Vitamin K_3_, 3.0 mg; Vitamin B_6_, 3.0 mg; Vitamin B_12_, 12 μg; Riboflavin, 4.0 mg; Thiamine, 1.5 mg; Niacin, 40 mg; Pantothenic acid, 15 mg; Folic acid, 0.7 mg; Biotin, 44 μg; choline chloride, 400 mg; copper, 10mg; iron, 90 mg; zinc, 80 mg; manganese, 30 mg; iodine, 0.35 mg; selenium, 0.3 mg.

**Table 2:** Effect of dietary VD_3_ and 25(OH)VD_3_ supplementation on the growth performance of weaned piglets.

Items	NC	PC	2000 IU VD_3_	2000 IU	SEM	*p*-Value
				25(OH)VD_3_		
**BW, kg**						
d 0	7.16	7.17	7.16	7.15	0.04	0.30
d 14	12.65	13.36	13.32	13.57	0.25	0.06
d 28	18.50 ^c^	19.94 ^b^	19.12 ^bc^	21.23 ^a^	0.29	<0.01
d 42	29.61 ^c^	31.54 ^b^	30.33 ^c^	33.20 ^a^	0.41	0.01
**d 0–14**						
ADG, g	318	354	353	378	9.29	0.19
ADFI, g	521	562	569	589	16.11	0.68
FCR	0.61	0.64	0.62	0.64	0.01	0.31
**d 14–28**						
ADG, g	418 ^c^	469 ^b^	411 ^c^	547 ^a^	13.81	<0.01
ADFI, g	733 ^b^	798 ^ab^	710 ^b^	887 ^a^	37.78	0.04
FCR	0.57	0.56	0.59	0.65	0.03	0.32
**d 28–42**						
ADG, g	794 ^b^	829 ^ab^	777 ^b^	860 ^a^	15.24	<0.01
ADFI, g	1526 ^b^	1482 ^c^	1528 ^b^	1656 ^a^	29.93	0.01
FCR	0.52	0.55	0.51	0.52	0.03	0.15
**d 0–42**						
ADG, g	510 ^c^	551 ^bc^	532 ^c^	590 ^a^	10.38	<0.01
ADFI, g	910 ^c^	944 ^bc^	936 ^bc^	1044 ^a^	26.31	0.01
FCR	0.56	0.58	0.56	0.56	0.02	0.43
**Diarrhea incidence %**						
d 0–14	5.42	4.5	4.89	5.29	0.86	0.82
d 14–28	6.48	2.25	4.5	2.12	0.64	0.86
d 0–28	5.95	3.37	4.7	3.70	0.71	0.89

Note: SEM is standard error of the mean. Different letters on the shoulder of the same row of data indicate significant differences (*p* < 0.05), n = 6. ADG, average daily weight gain; ADFI, average daily feed intake; FCR, feed conversion ratio; NC, negative control; PC: positive control; VD_3_, vitamin D_3_; 25(OH)VD_3_, 25-hydroxyvitamin D_3_.

**Table 3:** Effect of VD_3_ and 25(OH)VD_3_ on the skeletal performance of weaned piglets.

Items	NC	PC	2000 IU VD_3_	2000 IU 25(OH)VD_3_	SEM	*p*-Value
**Tibia**						
Bone mineral content (g)	4.58 ^b^	3.97^b^	5.48 ^a^	4.87 ^ab^	0.20	<0.01
Bone mineral density (g/cm^2^)	0.45	0.43	0.45	0.46	0.01	0.56
Breaking strength	1290 ^b^	1293 ^b^	1368 ^a^	1396 ^a^	26.20	0.03
Failure displacement	3.34	3.00	3.16	3.40	0.13	0.21
Stiffness(N/mm)	606.00	572.00	690.00	682.00	36.47	0.07
Absorbed energy(J)	2.82	2.46	2.84	2.73	0.18	0.72
Calcium (%)	18.62 ^b^	18.79 ^b^	19.41 ^b^	21.05 ^a^	0.39	0.01
Phosphorus (%)	9.20	9.72	10.00	9.96	0.31	0.35
**Femur**						
Bone mineral content (g)	4.60 ^ab^	3.88 ^b^	4.83 ^ab^	5.55 ^a^	0.28	0.01
Bone mineral density (g/cm^2^)	0.44	0.44	0.46	0.46	0.01	0.48
Breaking strength (N)	1251 ^c^	1269 ^c^	1398 ^b^	1436 ^a^	21.67	0.01
Failure displacement (mm)	3.39	3.35	3.42	3.62	0.20	0.64
Stiffness (N/mm)	616.00	604.00	683.00	694.00	38.51	0.08
Absorbed energy(J)	2.68	2.21	2.61	2.63	0.17	0.46
Calcium (%)	20.96 ^b^	21.14 ^b^	21.82 ^ab^	23.62 ^a^	0.57	0.02
Phosphorus (%)	10.59	11.16	11.47	11.42	0.44	0.52

Note: SEM is standard error of the mean. Different letters on the shoulder of the same row of data indicate significant differences (*p* < 0.05), n = 6. NC, negative control; PC, positive control; VD_3_, vitamin D_3_; 25(OH)VD_3_, 25-hydroxyvitamin D_3_. The bones were ash to calculate the concentrations of calcium and phosphorus.

## Data Availability

The data presented in this study are available on request from the corresponding author.

## Supplementary Materials

The following supporting information can be downloaded at: https://www.mdpi.com/article/10.3390/antiox11091750/s1, Table S1: Primer sequences used in RT-qPCR.
