# Supplementary material for: Dietary Supplementation of 25-Hydroxyvitamin D3 Improves Growth Performance, Antioxidant Capacity and Immune Function in Weaned Piglets
Source: Antioxidants (Basel). 2022 Sep 3;11(9):1750. doi: 10.3390/antiox11091750 (PMC9495450; doi:10.3390/antiox11091750)
Supplement: Supplementary file 1 [file antioxidants-11-01750-s001.zip › antioxidants-1815002-supplementary.pdf]

Table S1. Primer sequences used in RT-qPCR

Target genes		Primer sequence(5'→3')
ZO-1	Forward	GCCATCCACTCCTGCCTAT
	Reverse	CGGGACCTGCTCATAACTTC
Occludin	Forward	CAGCAGCAGTGGTAACTTGG
	Reverse	CAGCAGCAGTGGTAACTTGG
Claudin-1	Forward	AAGGACAAAACCGTGTGGGA
	Reverse	CTCTCCCCACATTCGAGATGATT
pBD-1	Forward	TGCCACAGGTGCCGATCT
	Reverse	CTGTTAGCTGCTTAAGGAATAAAGGC
pBD-2	Forward	CCAGAGGTCCGACCACTACA
	Reverse	GGTCCCTTCAATCCTGTTGAA
PG1-5	Forward	CGGAGCTGTGTGACTTCAAGGAGAA
	Reverse	TGCCGTCGCAACCGTCATCCT
SOD	Forward	GTTGGAGACCTGGGCAATGT
	Reverse	CGGCCAATGATGGAATGGTC
CAT	Forward	AGATACTCCAAGGCGAAGGTG
	Reverse	AAAGCCACGAGGGTCACGAAC
GSH-Px	Forward	GCGGGAGCAGGACTTCTACGA
	Reverse	CCCGATAGTGCTGGTCTGTGAA
GAPDH	Forward	TGGTGAAGGTCGGAGTGAAC
	Reverse	GGAAGATGGTGATGCGATTTC
